# Supplementary material for: Experimental Lagos bat virus infection in straw-colored fruit bats: A suitable model for bat rabies in a natural reservoir species
Source: PLoS Negl Trop Dis. 2020 Dec 15;14(12):e0008898. doi: 10.1371/journal.pntd.0008898 (PMC7771871; doi:10.1371/journal.pntd.0008898)
Supplement: S1 Text — (DOCX) [file pntd.0008898.s001.docx]

**S1 Text. Incidental lesions detected in bats from the experiment and the natural-infected bats.**

There were several lesions that we considered to be incidental. Bat 4 (10^4.1^ TCID_50_ group, died with rabies) had small foci of calcification scattered in the interstitium of the salivary gland. Bat 1 (10^4.1^ TCID_50_ group, died with rabies) had small areas of fibrosis within the myocardium. Bat 8 (10^3.1^ TCID_50_ group, died with rabies) had a few lymphocytes infiltrating the atrial myocardium. Bat 11 (10^2.1^ TCID_50_ group, died with rabies) had several mild acute hemorrhages in the ventricular myocardium and a subacute thrombus in a large blood vessel in the kidney. Bat 3 (10^4.1^ TCID_50_ group, euthanized with no detectable LBV infection) had a focal area of cardiomyocyte degeneration, characterized by loss of cross-striation, fragmentation and increased intercellular spaces. This lesion was likely caused by cardiac exsanguination during euthanasia. Bats 5, 12 and 16 (different dose groups, died with rabies [bat 12] or euthanized with no detectable LBV infection [bats 5 and 16]) each had a few random aggregates of small numbers of lymphocytes in the liver. Bat 10, 14, 18 (different dose groups, died with rabies [bats 10 and 14] or euthanized with no detectable LBV infection [bat 18]) had no clear lymphoid follicles in the spleen, while periarteriolar lymphoid sheaths were present. Seven bats from the experiment (nos. 3, 4, 5, 7, 12, 15, 17 and 19, different dose groups, died with rabies [bats 4, 7, 12, and 19] or with no detectable LBV infection [bats 3, 5, 15, and 17]) and the naturally-infected bat each had mild lymphoplasmacytic interstitial nephritis. Bat 5 additionally had distended tubuli with protein casts in the kidney.
