# Supplementary figures and images for: Experimental Lagos bat virus infection in straw-colored fruit bats: A suitable model for bat rabies in a natural reservoir species
Source: PLoS Negl Trop Dis. 2020 Dec 15;14(12):e0008898. doi: 10.1371/journal.pntd.0008898 (PMC7771871; doi:10.1371/journal.pntd.0008898)

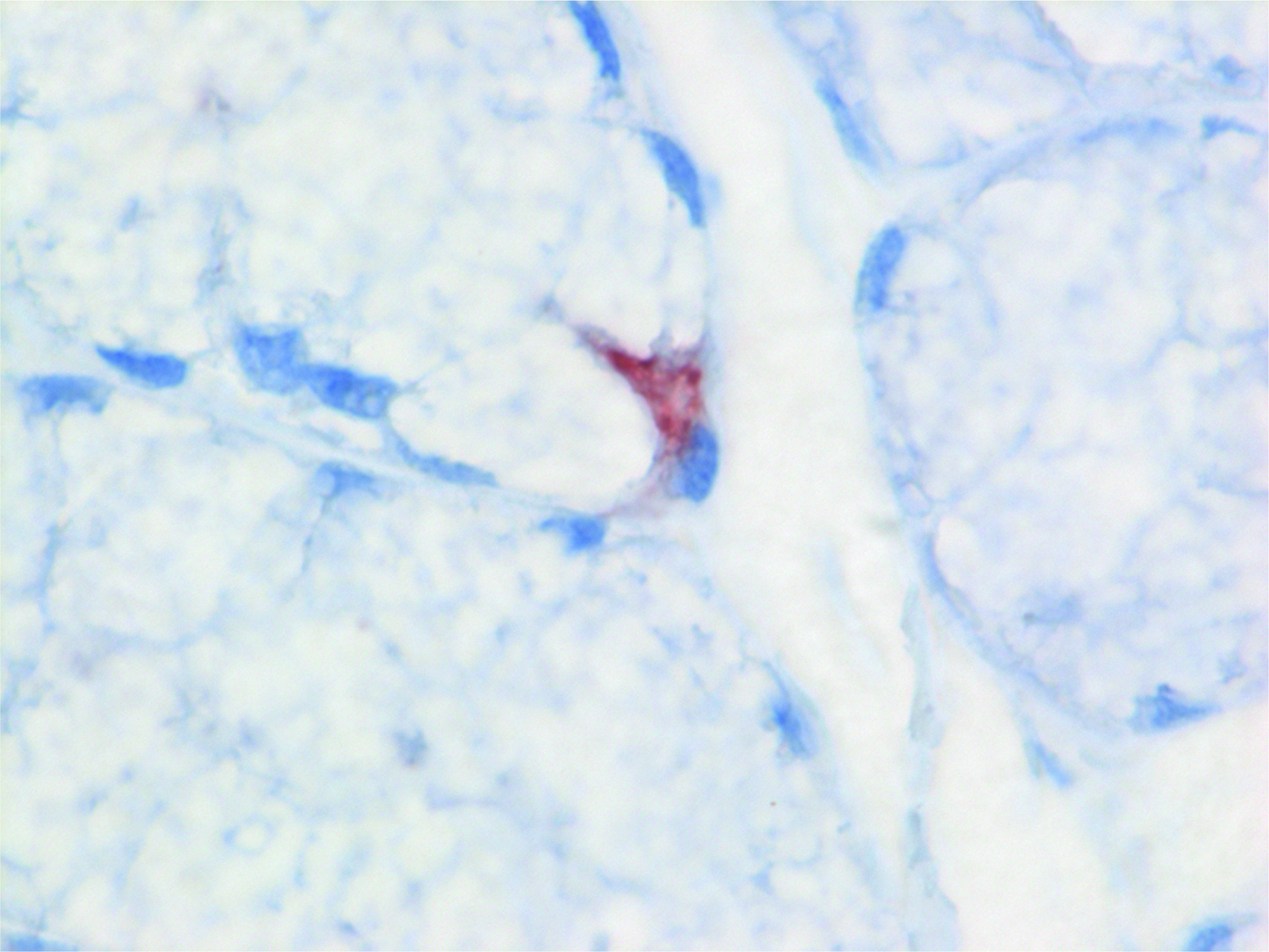

Supplement: S1 Fig — Mucous salivary gland acini separated by septa are shown. One of the lining myoepithelial cells expresses lyssavirus antigen (red granules). Lyssavirus IHC stain. Original magnification 100x objective. (TIF) [file pntd.0008898.s004.tif]

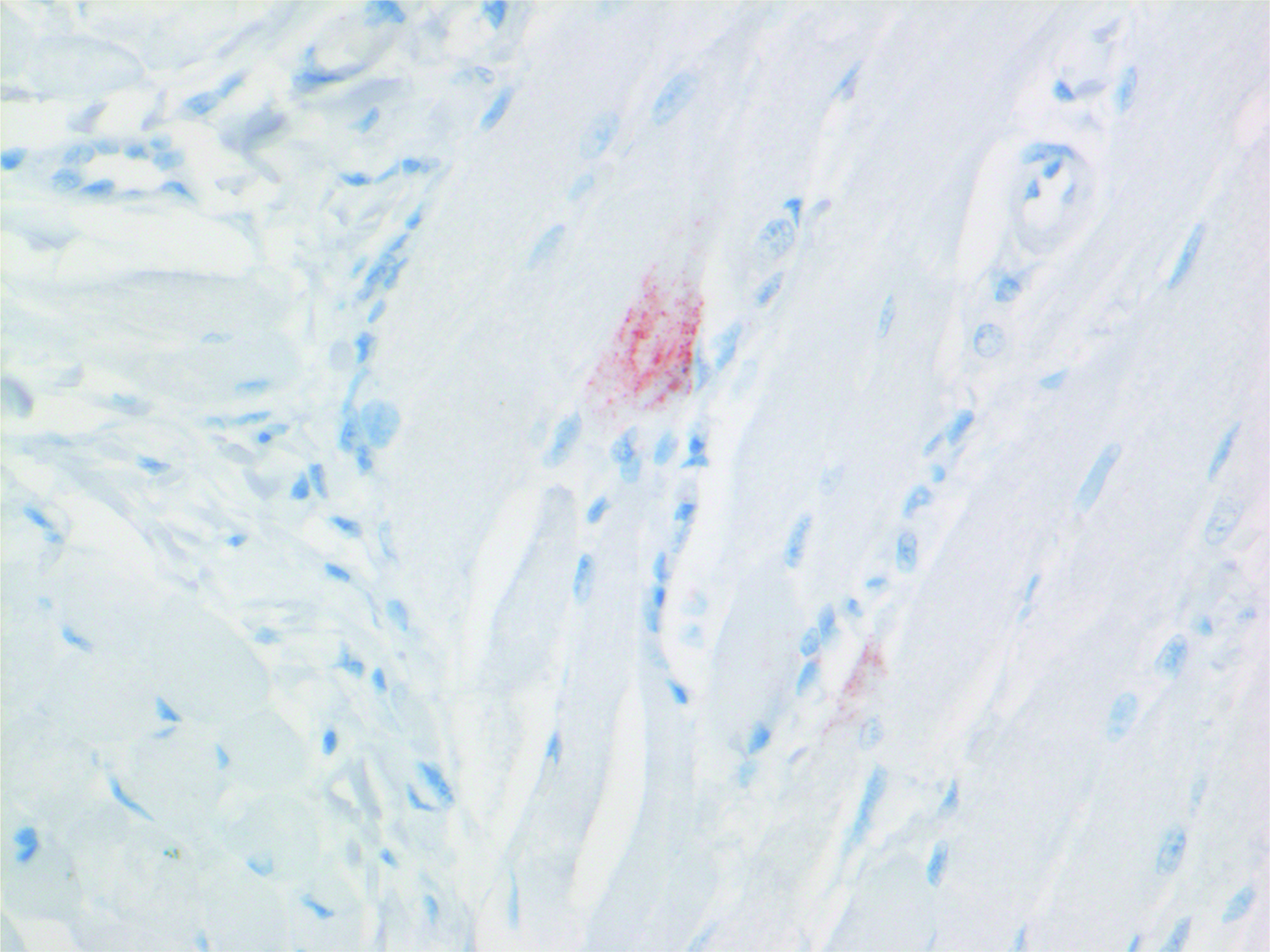

Supplement: S2 Fig — Antigen positive granules are interpreted as being localized in neuromuscular junctions, because the positive granules form discrete aggregates rather than being dispersed evenly throughout the cytoplasm of the myocytes. Lyssavirus IHC stain. Original magnification 40x objective. (TIF) [file pntd.0008898.s005.tif]
