# Supplementary material for: Experimental Lagos bat virus infection in straw-colored fruit bats: A suitable model for bat rabies in a natural reservoir species
Source: PLoS Negl Trop Dis. 2020 Dec 15;14(12):e0008898. doi: 10.1371/journal.pntd.0008898 (PMC7771871; doi:10.1371/journal.pntd.0008898)
Supplement: Attachment — Submitted filename: RebuttalPLOSntd LBV in EHII_final.docx [file pntd.0008898.s006.docx]

Dear Dr. Hughes and Dr. Blacksell,

Thank you for your invitation to submit a revised version of our paper. Hereby, in the text below we provide a detailed list of our responses to the reviewers’ comments and a description of the consequent changes we have made in the manuscript. For clarity, the reviewers’ comments are highlighted by making the text bold, while our replies to them are in regular font.

Kind regards,

Andrew Cunningham

Responses to reviewer #1:

**REVIEWER: The background section referenced key literature in the field, but could use updating to include more recent syntheses and context in the field of bat rabies (e.g. doi: 10.1016/B978-0-12-818705-0.00007-8). Please find some minor suggestions below which may improve clarity.**

REPLY: We thank the reviewer for pointing out the suggested literature and we have incorporated these references as suggested.

**REVIEWER: L79-81 – please also consider this recent example of suspected natural transmission in a captive setting however – doi: 10.7589/2019-04-108**

REPLY: The reference was added to the manuscript. Indeed, it describes an interesting side-result from this experimental study.

**REVIEWER: L147 – please identify the units for value “27”**

REPLY: Since antibody concentration is given as the reciprocal of a dilution, it has no scientific unit. To make the value more clear we have changed (highlighted here for clarity): 'However, the highest antibody titer measured in these two bats was 27...‘ to current manuscript line 147 'However, the highest **reciprocal** antibody titer measured in these two bats was 27...' All neutralizing antibody levels were expressed as reciprocal titers, which were calculated using the Spearman–Karber method.

**REVIEWER: L149-150 – please clarify that the VNA were detected from serum samples**

REPLY: To indicate clearly on which type of samples VNA’s were performed we have made two adjustments in the text (highlighted below for clarity):

1. ‘Four of the six experimentally-inoculated bats that developed LBV-neutralizing antibodies were in the two highest virus dose groups…’ changed to ‘ Four of the six experimentally-inoculated bats that developed LBV-neutralizing antibodies **in their serum** were in the two highest virus dose groups…’ in lines 140 and 141 of the current manuscript.
2. ‘In the naturally-infected bat used in this study no LBV-neutralizing antibodies had been detected using the modified rapid fluorescent focus inhibition test.’ changed to ‘In the naturally-infected bat used in this study no LBV-neutralizing antibodies had been detected **in serum** using the modified rapid fluorescent focus inhibition test.’ In lines 149 and 150 of the current manuscript.

**REVIEWER: L276-279 – it seems the authors might also consider and discuss the possibility of defective interference particles (see PMID: 833940). The middle range doses may have fewer DI particles than higher doses, somewhat consistent with serology?**

REPLY: We aimed to keep the level of DI particles as low as possible by making a virus stock grown up from a low dose inoculum and with minimal passage in cell culture. We used the same virus stock (diluted to different final concentrations) for the different groups, so do not expect a different ratio of infectious virus particles to DI particles.

**REVIEWER: L296-301 – I think the authors may also consider for discussion the potential for differences in isolate pathology or clinical “phenotype” depending on whether it was sourced from brain versus salivary gland material of the naturally infected host?**

REPLY: We agree with the reviewer that we cannot exclude that this might have played a role. We did discuss this later, in the current manuscript, lines 372-377 ‘Possible reasons for a lack of salivary gland epithelium infection include the bats dying or being euthanized before the infection could reach the salivary gland epithelial cells, and an inability of our LBV strain to infect salivary gland epithelial cells.’ In our revised version we now added here: ‘Some authors (e.g., Charlton, 1987; Gilbert, 2020) used viruses isolated from the oral cavity instead of from the brain for experimental infection studies; this may ensure that viruses are from a population that is able to infect cells important for excretion.’

**REVIEWER:**

**Methods – page 29.** **Please provide units for the number 15.57. If this is a VNA titer, I wondered whether the testing was really precise enough to estimate to two decimal places (hundredths of unit)?**

REPLY: Agreed, we have rounded the number (Line 512 on page 24 of the current manuscript). As above, there is no unit for a reciprocal antibody titer.

**REVIEWER:**

**S1 Table – if all of the study subjects were captive bred, it may be interesting to see and statistically evaluate continuous host age (years, months) data?**

REPLY: Age class was based on the microchip number and approximate date of birth. Unfortunately, due to logistical constraints on the frequency (approx. quarterly) of catching-up all of the captive bats and microchipping pups/juveniles, it is not possible to know their exact date of birth.

Current lines 132-134 on page 7 reporting the lack of statistical correlations with age class, did not change: ‘There was no correlation between age (S1 Table) and infection status [χ2 (1, N = 20) = 0.90, p = .34], or between age and the development of antibodies [χ2 (1, N = 20) = 1.62, p = .20, an alpha level of .05 was used].’

Responses to reviewer #3:

**REVIEWER:**

**1.	In general the authors use very outdated references especially in the introduction of the manuscript. This must be improved and replace with more up to date references.**

**Eg. Ref 1 is a 2017 paper. More lyssavirus species have been identified in recent years. Rather use ICTV official reference. Hu et al., 2018; Amarasinghe et al., 2019**

REPLY: We thank the reviewer for taking the effort to make this specific suggestion, and we have replaced the outdated reference with the one suggested. Also, we have added additional up-to-date references elsewhere in the manuscript; for example, those suggested by Reviewer #1.

**REVIEWER: Also specifically revise ref 3-6, 17, 8, 47 to name a few.**

REPLY:

Original references 3-6 have been replaced by Pieracci et al, 2019 and Shipley et al, 2019

Original reference 17 Markotter W, Kuzmin I, Rupprecht CE, Randles J, Sabeta CT, Wandeler AI, et al. Isolation of Lagos bat virus from water mongoose. Emerg Infect Dis. 2006;12: 1913-1918: We think this reference is still relevant to show that Lagos bat virus has been shown to be capable of infecting a species of terrestrial carnivore.

Original reference 8: Freire de Carvalho M, Vigilato MAN, Pompei JA, Rocha F, Vokaty A, Molina-Flores B, et al. Rabies in the Americas: 1998-2014. PLoS Negl Trop Dis. 2018;12:e0006271. Epub 2018/03/21.

doi: 10.1371/journal.pntd.0006271PNTD-D-17-00692

This is a publication from 2018 including data up to 2014. There has not been any updated information published since then, to the best of our knowledge. Because of the order, we think ‘8’ might have been a typo, and that the reviewer had meant to refer to ‘18’ (study from 1992).

18, like 17 is still a relevant reference to show Lagos bat virus can infect dogs. We have not been able to find any additional recent references to substantiate this statement.

**REVIEWER: Two recent reviews have been published that can also be considered:**

**Markotter et al., 202Bat-borne viruses in Africa…. doi:10.1111/jzo.12769**

**Markotter W and Coertse J. Bat lyssaviruses. OIE technical report. Rev. Sci. Tech. Off. Int. Epiz., 2018, 37 (2), 385-400**

REPLY: We thank the reviewer for taking the effort to make specific suggestions for additional literature. They were considered and we have now added Markotter et al, 2020.

**REVIEWER**

**2.	I suggest the authors introduce Lagos bat at the start of the introduction and where it fits into lyssavirus taxonomy and epidemiology**

REPLY: The taxonomy of the strain of Lagos bat virus used in our study was reported by Freuling et al, 2015 (Freuling CM, Binger T, Beer M, Adu-Sarkodie Y, Schatz J, Fischer M, et al. Lagos bat virus transmission in an Eidolon helvum bat colony, Ghana. Virus Research. 2015;210: 42-45.). To make this clearer for readers especially interested in this, we have now added this reference in current line 104. The main goal of our study is to develop of an experimental model that mimics natural infection in a natural reservoir bat host (current lines 101 and 102). Therefore, we feel it is more appropriate to first start the introduction covering why this is necessary and then, after this, to introduce Lagos bat virus.

**REVIEWER**

**3.	Line 98: I do not agree that LBV will be a major public health threat. Yes we may miss/see a few sporadic cases but not “major threat”. Please revise**

REPLY: In current lines 97 and 98 we have changed ‘… this pathogen has the potential to **be an important public health threat**’ to ‘‘… this pathogen has the potential to **threaten public health’**.

**REVIEWER**

**4.	Line 133 and Supplementary S1 refer to age of the bats and a distinction is made between adult and sub adult. How was this distinction determined? The method of defining these age classes may influence the lack in correlation between age classes and infections status.**

REPLY: Age class was based on the microchip number and approximate date of birth. Unfortunately, due to logistical constraints on the frequency (approx. quarterly) of catching-up all of the captive bats and microchipping pups/juveniles, it is not possible to know their exact date of birth.

**REVIEWER**

**5.	Line 175 and methodology: The authors state that all oral swabs tested negative. This is very unexpected since virus was detected in the tongue. Did the authors perform additional controls to determine if extractions of nucleic acids from the swabs were successful?**

REPLY: We agree that the lack of detection of virus in any of our 97 swabs tested is unexpected, it remains a mystery to us.

As a control for RNA isolation, beta-actin was tested for along with the Lagos bat virus RT-PCR. For 14 of the 97 swabs (14%), beta actin was not detected. Similarly, to our study, in which 86% of the oral swabs tested positive for beta-actin, in another study 75%, 94% and 100% of oral swabs tested positive for beta-actin [Smiley Evans T, Barry PA, Gilardi KV, et al. Optimization of a Novel Non-invasive Oral Sampling Technique for Zoonotic Pathogen Surveillance in Nonhuman Primates. PLoS Negl Trop Dis. 2015;9(6):e0003813. Published 2015 Jun 5. doi:10.1371/journal.pntd.0003813]. Our results might suggest that the quality of samples was suboptimal for those 14, but this could only be a small part of the explanation for the negative swabs.

As it might help to understand, at least partly, the lack of virus detection in our oral swabs, we added the lack of beta-actin detection in the oral swabs in our methodology (lines 543 and 543 on page 25), results (line 180 on page 9) and in the discussion (lines 410-412: here we state ‘Our lack of detection of beta actin in 14 (86%) of our samples, might suggest RNA extraction failed, but this would explain the lack of detection of lyssavirus in only a minor proportion of our samples.’). As this is an important result and is unexpected, we have also added further possible explanations for this in the text (lines 412-424) – see also response below.

**REVIEWER: Furthermore although swabs were collected regularly in the study, the authors decide to only test swabs of bats that survived at the end of the study (not all swabs were tested). This is a major limitation and I suggest the authors include these results. See also point 8.**

REPLY: There appears to be a misunderstanding here, so we have amended the text to clarify what was done. We tested all oral swabs collected from those bats that did *not* survive to the end of the study. This is explained in the methodology in the current line 495 to 497 on page 24: ‘Swabs were tested for the presence of virus RNA in the following ways: (1) if the bat died during the course of the experiment, all swabs available were tested; (2) if the bat survived until the end of the experiment, only the oral swabs taken at the time of necropsy were tested.’ To make this clearer in the results section, we have added the following sentence in current lines 177-179 on page 9 ‘All oral swabs taken from bats that died during the study were tested, as well as the oral swabs taken on the day of termination of the study from healthy bats that survived to the end of the study.’

We did not test earlier oral swabs taken from the bats that failed to become sick and which survived the experiment as a productive infection needs to go via the brain and a bat with a lyssavirus brain infection will show clinical signs or death (Begeman L, GeurtsvanKessel C, Finke S, Freuling CM, Koopmans M, Müller T, et al. Comparative pathogenesis of rabies in bats and carnivores, and implications for spillover to humans. Lancet Infect Dis. 2018;18: e147-e59).

**REVIEWER:**

**On page 20 the authors also mention that the unexpected negative swab results may be due to technical issues and they refer to a previous paper. I suggest summarizing the issues here also since these negative results are unexpected.**

REPLY: We have now added substantially to the text from current line 412: ‘There are several other possible explanations for the lack of detection of viral excretion, as outlined previously by Suu-Ire et al (2018). First, IHC detection of lyssavirus antigen in oral tissues may be more sensitive than hn-RT-PCR detection of lyssaviral RNA in oral swabs. Second, although there was virus in tongue surface epithelium, there might have been no excretion into the oral cavity, although this does not fit well with what is known of the pathogenesis of other lyssaviral infections {Begeman, 2018}. Third, it might be that virus excretion was intermittent and oral swabs were taken at a time when virus was not being excreted. Intermittent excretion has been proposed to explain alternating positive and negative results of serially collected oral swabs in other experimental lyssavirus infections in bats {Baer, 1967}. However, virus was not detected by hn-RT-PCR in any of our oral swabs. Fourth, there may have been loss or degradation of viral RNA in oral swab samples during transport or processing. LBV has been detected in an oral swab of a naturally infected straw-colored fruit bat previously {Kuzmin, 2008}, showing that this is technically possible.’

**REVIEWER**

6.	**Line 261 – Expand on reasons why a longer incubation period is associated with lower viral doses.**

REPLY: We have now expanded on this section, from the current line 270: ‘} …’, the reasons for which are not clear. A possible explanation is that a low initial virus dose results in a small number of initially infected neurons, which consequently lowers the number of neurons infected in the brain in subsequent rounds of interneuronal transmission of virus, and in this way increases the time to development of clinical signs.’

**REVIEWER**

**7.	Line 277 – The authors refer to the “Goldilocks” principle several times in the publication and this was also previously published. However not all readers will understand this context and I suggest the authors rather just explain the principle.**

REPLY: We have slightly modified our explanation of the principle to make it clearer in current lines 286-287. We feel that by reiterating a previously-published and memorable name for this principle, it might stick better with the reader.

**REVIEWER**

**8.	Line 308-314 need to be revised. On the one hand the authors discussed that their study and others found that bats develop an immune response without clinical diseases and this occur without any viral excretion of the virus. This is the explanation for high seroprevelance observed. However immune response must be in response to an infection. This is contradictory.**

REPLY: In a slowly developing infection, as usually occurs with lyssavirus infection, it could be possible for an effective immune response to develop before that infection becomes widespread (e.g. reaches the brain), or productive (i.e. infectious virus is excreted). In our interpretation of the results of our study, therefore, we leave open the possibilities that early infection occurred and was abrogated, or that the exposure to virus alone caused an immune response to develop -current line 317 ‘there is no evidence that the virus had reached the brain before being cleared by the immune response’-.

**REVIEWER (continued)**

**How can high seroprevalence be observed, if no excretion and very low prevalence of virus? How can a very low prevalence of virus and no excretion in most infections cause such high seroprevalence in bat populations that are known to be thousands and even hundreds of thousands large?**

REPLY:

We agree that it is a conundrum that infection prevalence is so low, yet seroprevalence is so high. We suggest that our results help to shed light on how this occurs in nature. In our interpretation of our results, we suggest that high seroprevalence in combination with low virus prevalence might occur when bats in roosts experience lyssavirus exposure in the absence of productive infection. This could be through exposure to high titers of virus (as in our high titer cohorts) or if multiple exposures to low virus titers (below a threshold of infectivity) occurred and stimulated high antibodies responses. For the virus to remain in the population it is not necessary that all exposed bats get a *productive* infection.

In current manuscript lines 324 to 328 we added: ‘We suggest that high seroprevalence in combination with low virus prevalence might occur when bats in roosts experience lyssavirus exposure in the absence of productive infection. This could be through exposure to high titers of virus (as in our high titer groups) or if multiple exposures to low virus titers occurred and stimulated high antibodies responses.’

**See point 5 again where I mentioned that the authors should have tested all swabs collected at different time points, also in the bats that survived, to ensure there was no viral excretion at any time point.**

REPLY: Please see our explanation about this point, above.

**REVIEWER**

**9.	Line 338-340. It is unclear how the results in this study directly relate to the management of human exposures to animals? The authors must please clarify this clearly what they practically mean by this statement. Since the correct management of post exposure decisions is crucial it should not be left to interpretation by the reader.**

REPLY: Agreed. We have made changes in the current lines 427 to 431 ’… , that the outcomes of infection in straw-colored fruit bats are, in general, not different from those for RABV infection in other species. Hence, people exposed to LBV should be treated. RABV vaccination and PEP are not likely to protect against LBV, so there is a need to develop treatments that do work for exposure to LBV and other phylogroup 2 lyssaviruses {Fisher, 2020}.’

**REVIEWER**

**10.	Methodology:**

**Virus preparation (page 22). Pleas provide more detail on the virus used e.g. specific lab number and Genbank accession number for sequence if available.**

REPLY: We have now added the following information in the methodology related to the virus strain used in this study in lines 467 and 468: GH 325, FLI lab. No.: 31225 {Freuling, 2015}, INSDC sequence databases (LN849915), GenBank: LN849915.1.

**REVIEWER:**

**Serology:** **Why did the authors use dog sera and not negative bat sera as a negative control. Non-specific reactions are known to be species specific.**

REPLY: Although the bats we used were captive-bred in a closed colony in which no bat has tested positive for LBV infection, we did not have access to true SPF bats. Given the absence of Lagos bat virus in Europe, we considered the use of European-sourced dog sera as a negative control to be reasonable.

**REVIEWER**:

**It is also not clear how the cut-off of 15.59 was determined for sero positivity. Please explain.**

REPLY: For the standard FAVN for rabies virus– the OIE serum control standard (0.5IU/ml) is used as a cut off. The average reciprocal titer for this serum is 15.59. Anything on the standard FAVN above this titer is able to neutralize, while a titer below this is insufficient to neutralize. Due to the lack of an ideal, standardized control for Lagos bat virus FAVN, and therefore the uncertainty in our cut-off, we wrote ‘were *considered* seropositive’ (lines 511 and 512). Also, in response to another reviewer, we have changed 15.59 to 16.
