# Supplementary material for: Experimental Lagos bat virus infection in straw-colored fruit bats: A suitable model for bat rabies in a natural reservoir species
Source: PLoS Negl Trop Dis. 2020 Dec 15;14(12):e0008898. doi: 10.1371/journal.pntd.0008898 (PMC7771871; doi:10.1371/journal.pntd.0008898)
Supplement: Attachment — Submitted filename: RebuttalPLOSntd LBV in EHII_new.docx [file pntd.0008898.s007.docx]

Dear Dr. Hughes and Dr. Blacksell,

Thank you for your friendly reply and the invitation to submit another revised version of our paper. Hereby, in the text below we provide a list of our responses to the three remaining reviewers’ comments and a description of the consequent changes we have made in the manuscript. For clarity, the reviewers’ comments are highlighted by making the text bold, while our replies to them are in regular font.

Kind regards,

Andrew Cunningham

**1) REVIEWER: L276-279 – it seems the authors might also consider and discuss the possibility of defective interference particles (see PMID: 833940). The middle range doses may have fewer DI particles than higher doses, somewhat consistent with serology?**

**PREVIOUS REPLY: We aimed to keep the level of DI particles as low as possible by making a virus stock grown up from a low dose inoculum and with minimal passage in cell culture. We used the same virus stock (diluted to different final concentrations) for the different groups, so do not expect a different ratio of infectious virus particles to DI particles.**

CURRENT REPLY:

We added in lines 309-314 in the discussion section: ‘The inoculum could contain apart from infectious virus, defective interfering viral particles that trigger the development of an immune response [71]. We aimed to keep the level of defective interfering particles as low as possible by making a virus stock grown up from a low dose inoculum, and with minimal passage in cell culture. We used the same virus stock, diluted to different final concentrations, for the different groups. Therefore, we do not expect there to have been a different ratio of infectious virus particles to defective interfering particles in each inoculum.’ Ref 71 is the reference suggested by the reviewer (PMID: 833940).

**2) REVIEWER Line 133 and Supplementary S1 refer to age of the bats and a distinction is made between adult and sub adult. How was this distinction determined? The method of defining these age**

**classes may influence the lack in correlation between age classes and infections status.**

**PREVIOUS REPLY: Age class was based on the microchip number and approximate date of birth. Unfortunately, due to logistical constraints on the frequency (approx. quarterly) of catching-up all of the captive bats and microchipping pups/juveniles, it is not possible to know their exact date of birth.**

CURRENT REPLY:

We made the following changes in lines 492 and 493 in the methodology section:

We deleted: Captive-bred bats were microchipped and their approximate date of birth was recorded.

We added: Age class was based on the approximate date of birth as per the microchip number: all captive bats were caught-up quarterly and any new pups or juveniles were microchipped.

**3) REVIEWER: Serology: Why did the authors use dog sera and not negative bat sera as a negative control. Non-specific reactions are known to be species specific.**

**PREVIOUS REPLY: Although the bats we used were captive-bred in a closed colony in which no bat has tested positive for LBV infection, we did not have access to true SPF bats. Given the absence of Lagos bat virus in Europe, we considered the use of European-sourced dog sera as a negative control to be reasonable.**

CURRENT REPLY:

In current lines 525-527:

We changed ‘Dog sera from unvaccinated healthy European dogs were used as negative controls.’ into: ‘We did not have access to true specific pathogen free bat sera, and as Lagos bat virus is absent in Europe, we used dog sera from unvaccinated healthy European dogs as negative controls.’
